# Identification and characterization of an Endo-glucanase secreted from cellulolytic *Escherichia coli* ZH-4

**DOI:** 10.1186/s12896-019-0556-0

**Published:** 2019-08-27

**Authors:** Jian Pang, Junshu Wang, Zhanying Liu, Qiancheng Zhang, Qingsheng Qi

**Affiliations:** 1 School of Chemical Engineering, Inner Mongolia University of Technology, Hohhot, 010051 Inner Mongolia China; 2 State Key Laboratory of Microbial Technology, Shandong University, Qingdao, 266237 China; 3 Inner Mongolia Energy Conservation and Emission Reduction Engineering Research Center in Fermentation Industry, Hohhot, 010051 China

**Keywords:** Cellulolytic *Escherichia coli* ZH-4, Secretory endo-glucanase, BcsZ, Enzyme characterization

## Abstract

**Background:**

In the previous study, the cellulolytic *Escherichia coli* ZH-4 isolated from bovine rumen was found to show extracellular cellulase activity and could degrade cellulose in the culture. The goal of this work was to identify and characterize the secreted cellulase of *E. coli* ZH-4. It will be helpful to re-understand *E. coli* and extend its application in industry.

**Results:**

A secreted cellulase was confirmed to be endo-glucanase BcsZ which was encoded by *bcsZ* gene and located in the cellulose synthase operon *bcsABZC* in cellulolytic *E. coli* ZH-4 by western blotting. Characterization of BcsZ indicated that a broad range of pH and temperature tolerance with optima at pH 6.0 and 50 °C, respectively. The apparent Michaelis–Menten constant (K_m_) and maximal reaction rate (V_max_) for BcsZ were 8.86 mg/mL and 0.3 μM/min·mg, respectively. Enzyme activity of BcsZ was enhanced by Mg^2+^ and inhibited by Zn^2+^, Cu^2+^ and Fe^3+^. BcsZ could hydrolyze carboxymethylcellulose (CMC) to produce cello-oligosaccharides, cellotriose, cellobiose and glucose.

**Conclusions:**

It is confirmed that extracellular cellulolytic capability of *E. coli* ZH-4 was attributed to BcsZ, which explained why *E. coli* ZH-4 can grow on cellulose. The endo-glucanase BcsZ from *E. coli*-ZH4 has some new characteristics which will extend the understanding of endo-glucanase. Analysis of the secretion characteristics of BcsZ provided a great reference for applying *E. coli* in multiple industrial fields.

## Background

Cellulose biomass is the most abundant carbohydrate on the earth. It can be hydrolyzed to reducing sugars for production of biofuels and chemicals, and thus has a great economic and commercial potential [1]. Cellulose as the main component of plant cell wall consists of linear long chains of β-1, 4 glucose units. Hydrolyzing cellulose by cellulase is ideal and promising for its utilization in environmentally friendly and high efficiency manner [2]. However, the cooperative action of three kinds of cellulolytic enzymes (endo-glucanase, exo-glucanase, and β-glucosidase) is essential in hydrolysis of cellulose to glucose [3]. Among three kinds of cellulolytic enzymes, endoglucanases plays an important role in the process of cellulose hydrolysis because it hydrolyzes the glycosidic bond randomly and shorten the cellulose chains in the initial stage of cellulose breakdown [4]. Cellulase is produced by various cellulolytic bacteria and fungi which have been isolated from different environment [5]. Isolating cellulolytic microorganisms from various environment and characterizating their cellulase are crucial for understanding the evolution mechanism of cellulolytic microorganisms and the hydrolysis mechanism of cellulase, which will promote their application in industry. In recent years, cellulase from bacteria was focused again because the glycoside hydrolases of cellulolytic bacteria are very diverse [6].

The previous study revealed that a cellulase (Cel-CD) from *Bacillus sp*. can be secreted into culture medium when Cel-CD was overexpressed in *E.coli* with or without its signal peptide, which indicated that *E. coli* has the capacity of secreting cellulase [7]. In addition, cellulolytic *E. coli* will be got when expressed this cellulase in *E. coli*, and the *E. coli* has a potential application to produce enzymes and chemicals directly from lignocellulose biomass [8].

In our previous study, a cellulolytic *E. coli* ZH-4 was isolated from the rumen [9]. *E. coli* ZH-4 is capable of converting corn straw to ethanol and hydrogen anaerobically. Extracellular endo-glucanase and β-glucosidase activity were detected. The results indicated that such enzymes were expressed and secreted in cellulolytic *E. coli* ZH-4. Genome sequence analysis of *E. coli* ZH-4 revealed an endoglucanase gene (Genbank accession number KY965823) encoding a BcsZ homolog.

From another point of view, cellulose is a major structural component in bacteria, which provides cell-surface and cell–cell interaction in various of biofilm models, and protects cells against chlorine treatment [10–12]. The previous study showed that inactivation of BcsZ altered the cellulose-associated phenotypes in *Salmonella enterica* serovar Typhimurium, such as rdar biofilm morphotype, cell clumping, biofilm formation, pellicle formation and flagella-dependent motility [10]. The hydrolase activity of BcsZ is hypothesized to mediate alignment of each β-1, 4 -glucan for proper cellulose microfibril formation [13]. *BcsZ* is a conserved component of the cellulose synthase operon *bcsABCZ,* which encodes the cellulose synthase BcsAB and the outer membrane porin for cellulose translocation and secretion [10, 14]. BcsZ belongs to Glycoside Hydrolase family 8 with endo-1,4-D-glucanase activity. BcsZ hydrolyzes glycosidic bonds by a pair of acidic residues inverting the anomeric configuration at the new reducing end [15]. The crystal structure analysis of BcsZ from *E. coli* showed an (α/α) _6_-barrel fold. BcsZ binds 4 glucan moieties of cellopentaose via highly conserved residues exclusively on the non-reducing side of its catalytic center [13]. However, whether BcsZ is responsible for cellulolytic ability of *E. coli* ZH-4 is uncertain. Little is known about the characteristic of BcsZ-ZH-4. Enzymatic hydrolysate of BcsZ-ZH-4 from cellulose is unknow.

In this study, the cellulolytic ability of *E. coli* ZH-4 was verified from BcsZ. The endoglucanase was assessed through transcription, expression and secretion. BcsZ from *E. coli* ZH-4 was functionally expressed in *E. coli* BL21 (DE3), and the recombinant protein was purified and characterized.

## Results

### Identification and verification of secreted cellulase in cellulolytic *E. coli* ZH-4

**Fig. 1:**
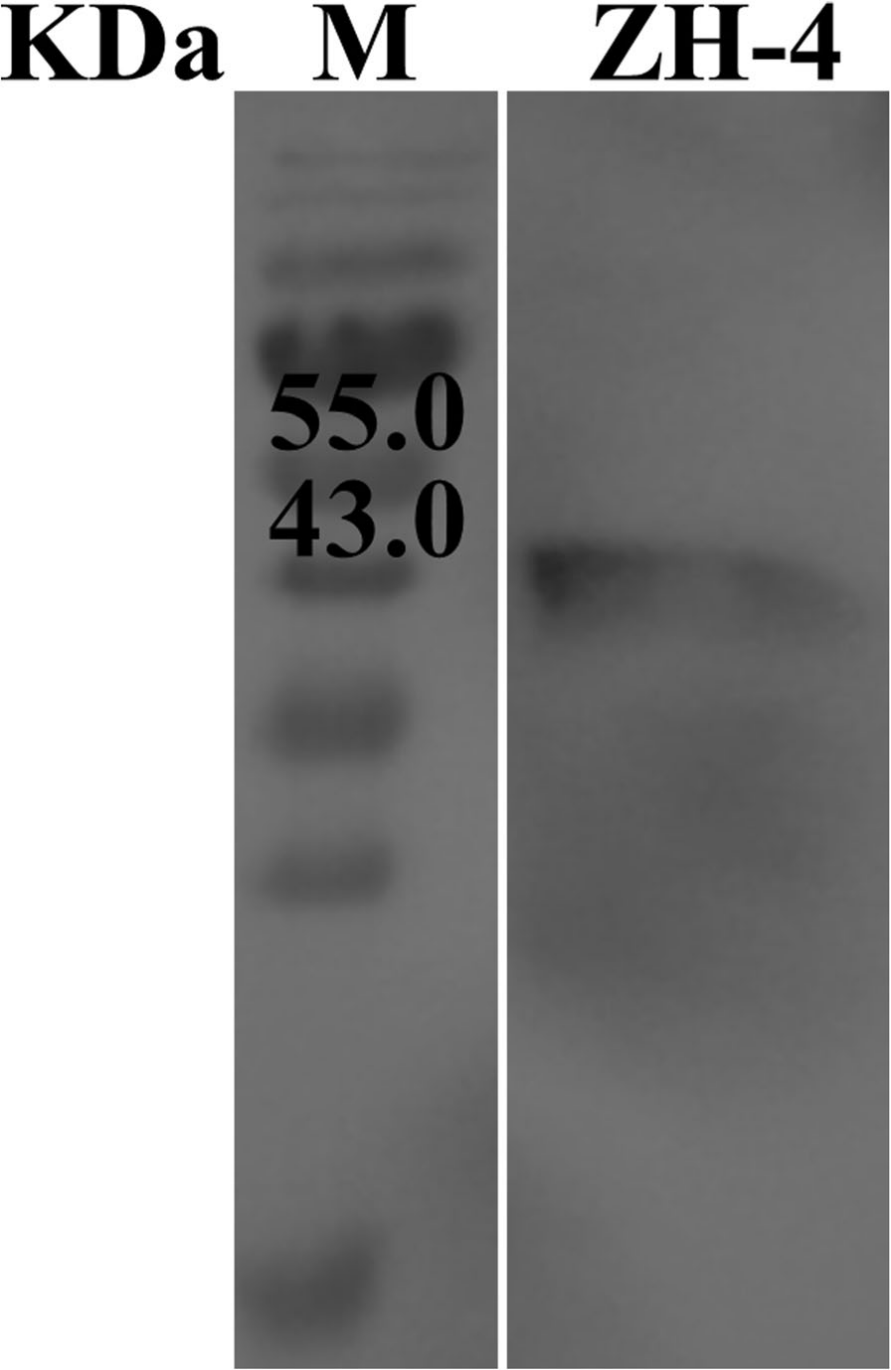
Western Blotting analysis of BcsZ in culture medium

**Fig. 2:**
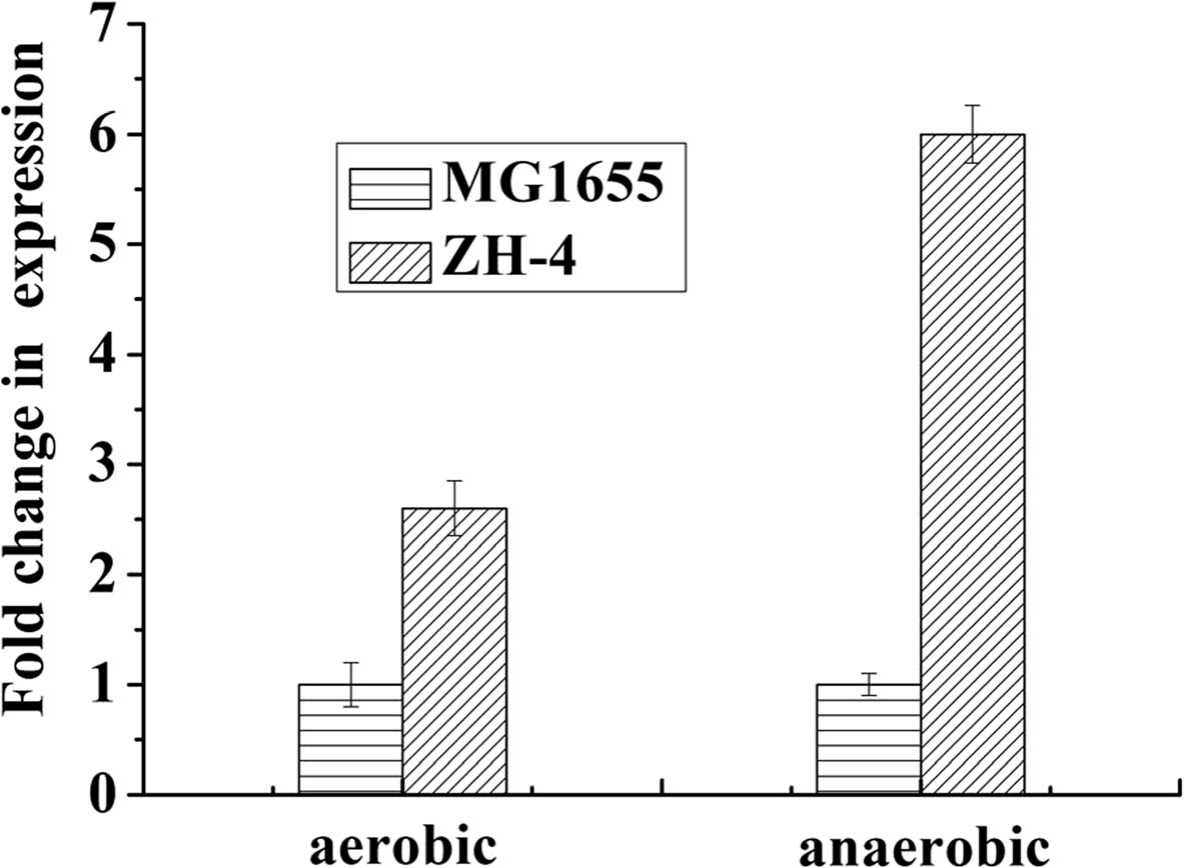
The fold change in gene expression of *bcsZ* in *E. coli* ZH-4 and MG1655

The mature protein in the culture medium was analyzed through western blotting to identify the extracellular endo-glucanase in ZH-4. The coding gene was located on the operon of *bcsABZC*. Meanwhile, the extracellular protein was verified by western blotting using BcsZ antiserum (Fig. 1). The transcription level of *bcsZ* in cellulolytic *E. coli* ZH-4 was also found to be 2.6 ± 0.25 and 6.0 ± 0.26 fold higher than that of *E. coli* MG1655 under aerobic and anaerobic condition respectively (Fig. 2).

A signal peptide sequence of 1–21 amino acid residues was found in *E. coli* ZH-4 by the analysis of the protein sequence. In compared to *E. coli* MG1655, there were two amino acids difference in BcsZ of *E. coli* ZH-4: Ser63 to Phe (Ser-Phe) and Ala71to Val (Ala-Val). The *bcsZ* located in the downstream of *bcsB* and was supposed under the control of the *bcsB* promoter. DNA-binding transcriptional dual regulator FNR (Fnr) regulates *bcsBZ* operon expression under anaerobiosis, and the putative FNR-binding site was identified in upstream of this operon. Genetic analysis of the operon indicated that there was no difference with that of MG1655 in the regulation and transcription region.

### Expression and purification of endo-glucanase BcsZ

**Fig. 3:**
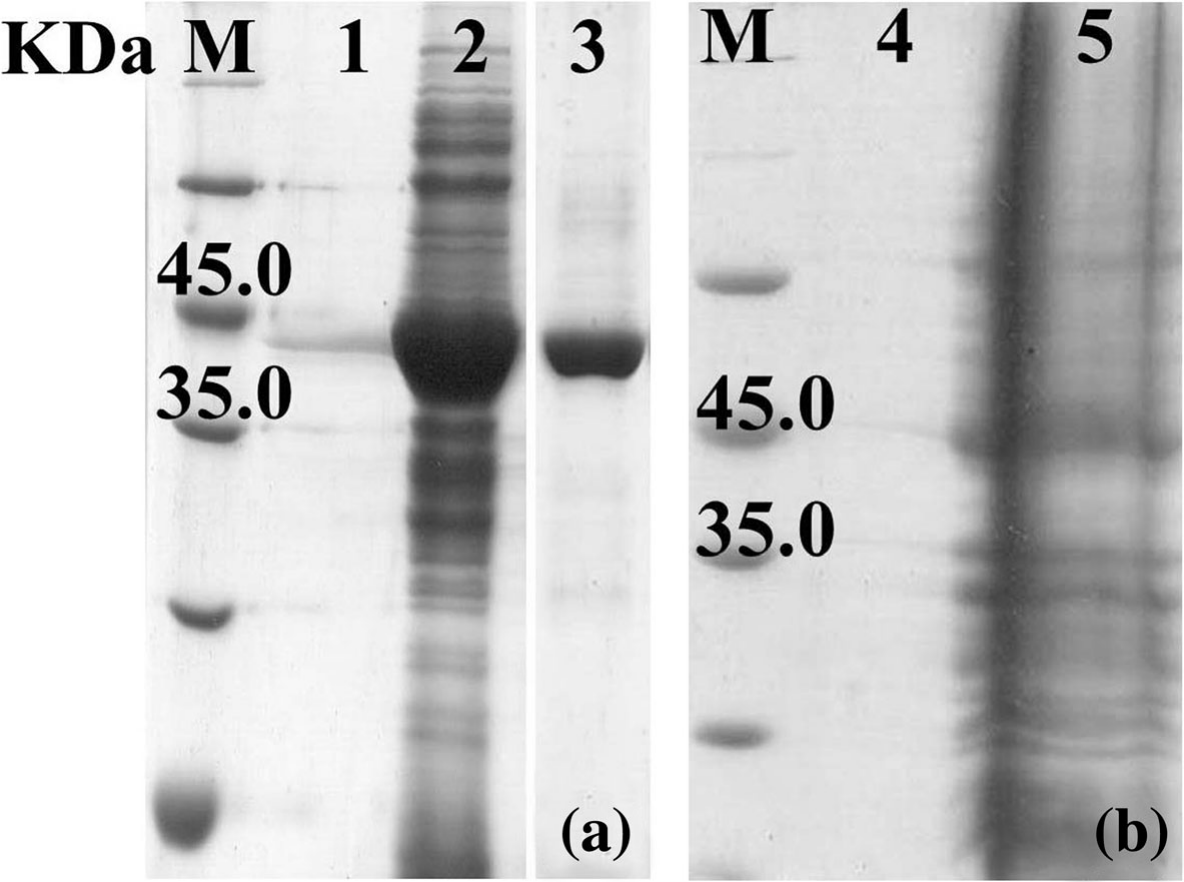
SDS–PAGE analysis of recombinant BcsZ protein stained with coomassie blue (**a**). M: Protein molecular weight marker; Lane 1: BcsZ in culture medium; Lane 2: BcsZ in cells; Line 3: The purified BcsZ. The *E. coli* BL21 (DE3) carrying the empty plasmid was used as control (**b**). Line 4: culture medium; Line 5: cells

The *bcsZ* gene amplified from *E. coli* ZH-4 was cloned in pET-28a vector, and then overexpressed in *E. coli* BL21 (DE3). BcsZ was detected in culture medium (Fig. 3a, line 1) with the recombinant cell by Sodium dodecyl sulfate polyacrylamide gel electrophoresis (SDS-PAGE). The result indicated that endo-glucanase, BcsZ, can be secreted to the outside of cell. The crude protein of BcsZ was purified, and the purified protein appeared as a single protein band on SDS–PAGE gel with a molecular mass of 41.7 KD, which was consistent with prediction (Fig. 3a, line 3). The *E. coli* BL21 (DE3) carrying the pET-28a vector (empty) was used as control (Fig. 3b).

### Characterization of the endo-glucanase BcsZ

**Fig. 4:**
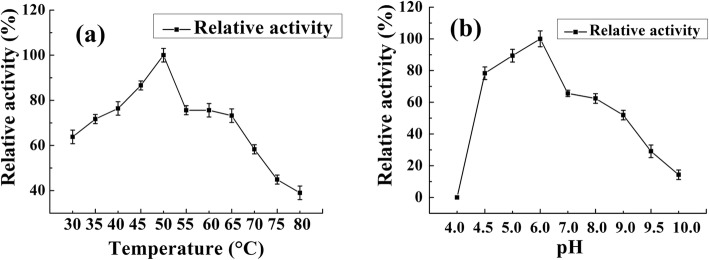
The optimum temperature (**a**) and pH (**b**) of BcsZ

The optimal temperature and pH of purified BcsZ were determined. As shown in Fig. 4a, the optimum temperature was 50 °C. With the rising of the temperature, the enzyme activity began to decrease, but retained over 70% at the temperature of till 65 °C. The enzyme activity sharply decreased when the temperature is over 65 °C, and was remained by 40% when the temperature was 80 °C. From Fig. 4b, the BcsZ displayed optimal activity at pH 6.0. It had relatively high enzyme activity at the broad pH range of 4.5–9.0. BcsZ was sensitive to the acidic condition. It remained about 80% activity when pH at 4.5, and enzyme activity was not detected when the pH was 4.0. The BcsZ protein has a tolerance to alkali solution because it retained over 15% of the maximum enzyme activity when the pH was 10.0.

**Fig. 5:**
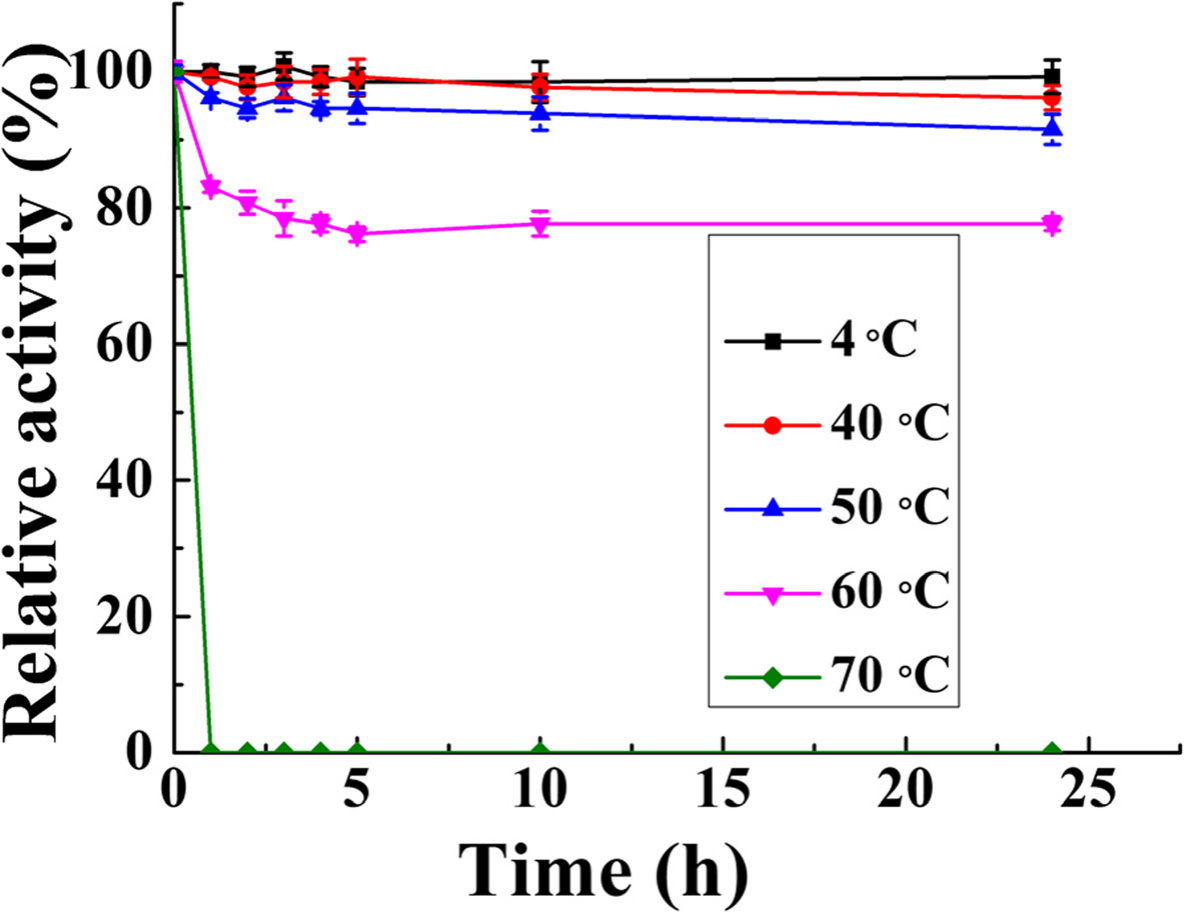
The thermostability of BcsZ

The thermal stability result was shown in Fig. 5, and showed BcsZ displayed high thermal stability at 50 °C and 60 °C with about 90 and 70% of its enzyme activity remained, respectively. The enzyme activity wasn’t detected at 70 °C after 0.5 h incubation.

The substrate specificity results were shown in Table 1. As shown in Table 1, BcsZ displayed enzyme activity with CMC, RAC and glucan (data not shown) as substrate. The enzyme showed a weak catalytic activity on Avicel, xylan, cellobiose, laminarin and chitin. The K_m_ value of BcsZ for CMC as substrate was 8.86 mg/mL, and the calculated V_max_ was 0.3 μM/min·mg.

**Table 1:** Substrate specificity of BcsZ

Substrate	Specific activity (IU/mg)
CMC	0.84 ± 0.05
RAC	0.52 ± 0.08
Avicel	< 0.01
xylan	< 0.01
cellobiose	< 0.01
laminarin	< 0.01
chitin	< 0.01

### Effect of metal ion on BcsZ activity

**Fig. 6:**
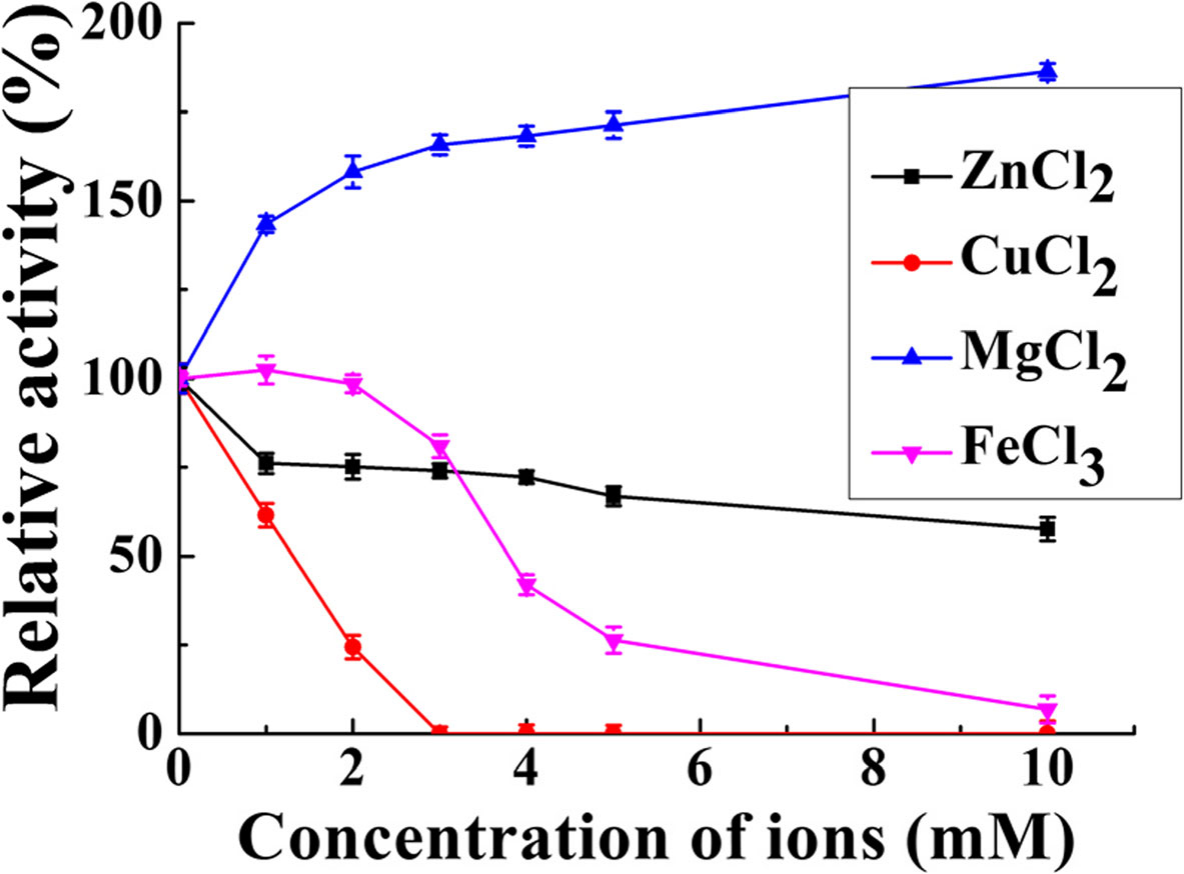
The effect of metal ions on the activity of BcsZ

The effect of metal ions on the enzyme activity of BcsZ was examined (Fig. 6). The enzyme activity was improved 165% by Mg^2+^. Fe^3+^ inhibited BcsZ, and 74% enzyme activity was lost with10 mM Fe^3+^. Zn^2+^ also inhibited the enzyme of BcsZ, and there was 66% of enzyme activity was left with 10 mM Zn^2+^. The enzyme was strongly inhibited by Cu^2+^, there is no enzyme activity detected when Cu^2+^ concentration reached 5 mM in the reaction system.

### Analysis of hydrolysis products by TLC

**Fig. 7:**
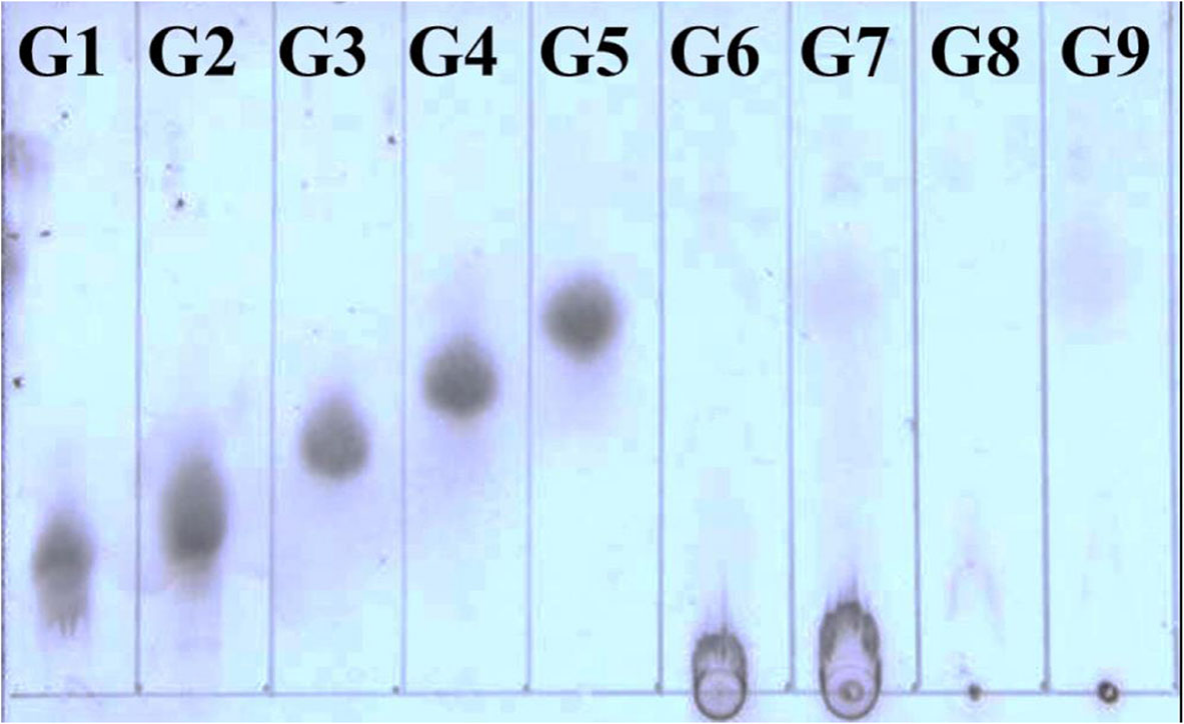
TLC analysis of hydrolysates of CMC and RAC catalyzed by BcsZ. Standards of Cellopentose (G1), Cellotetraose (G2), Cellotriose (G3), Cellobiose (G4) and Glucose (G5); CMC incubated with inactivated BcsZ as the control (G6), CMC incubated with BcsZ (G7); RAC incubated with inactivated BcsZ as the control (G8), RAC incubated with BcsZ (G9)

The hydrolysis products of CMC and RAC were analyzed by TLC. As shown in Fig. 7, glucose was released when BcsZ hydrolyzed RAC (G9). The hydrolysis products were glucose, a small amount of cellobiose and cellotriose, and unknown cello oligosaccharides from CMC (G7) as substrate.

## Discussion

In our previous study, a cellulolytic *E. coli* ZH-4 was isolated from bovine rumen, this strain could produce extracellular cellulases. In this study, secretion of BcsZ to culture medium was confirmed by western blotting (Fig. 1). These results explained the reason ZH-4 formed a clear zone in anaerobic Hungate roll tubes (containing cellulose and Congo red) [9]. While *E. coli* strain MG1655 and W3110 didn’t generate the above phenotype at the same condition. Secretion of cellulase (BcsZ) and its contribution to the cellulolytic capacity of *E. coli* ZH-4 were confirmed, which were consistent with our previous supposition and the published [9]. The previous studies showed overexpression of BcsZ in *E. coli* leads to its secretion to extracellular space and formation of clear zone on congo red plates [16, 17]. Further analysis confirmed that the transcription level of *bcsZ* in ZH-4 was higher than that of MG1655, especially under anaerobic condition. The increased transcription level of *bcsZ* is critical to the cellulolytic activity of *E. coli* ZH-4. Sequence analysis showed that the promoter, Fnr and FNR-binding site of *bcsZ* in *E. coli* ZH-4 is consistent with MG1655 and W3110. The genome of *E. coli* ZH-4 (5.3 Mb) is larger than *E. coli* MG1655 (4.6 Mb). It is supposed that some regulatory factors and elements regulate *bcsZ* transcription and expression in *E. coli* ZH-4. This may lead to the increased transcription level of *bcsZ* in *E. coli* ZH-4. Further elevated transcription level investigation of *bcsZ* may reveal the difference in BcsZ secretion between *E. coli* ZH-4 and MG1655.

The endo-glucanase BcsZ had the broad pH range and strong alkali tolerance. These characteristics of BcsZ might be affected by the rumen habitat and consistent with the previous reports. For instance, Gong et al. cloned and identified some novel hydrolase genes from a dairy cow rumen. The purified recombinant enzyme displayed optimal activity at pH 6.0 and 50 °C. It was stable over a broad pH range, from pH 4.0 to 10.0 [18]. Chang et al. reported an endoglucanase from yak rumen microorganisms and the optimal conditions for enzyme activity were 50 °C and pH 5.0 [19]. BcsZ is mesophilic enzyme which is in line with the other endoglucanse in some microorganisms such as *Bacillus sp.* HSH-810 [20], *Komagataeibacter xylinus* [21].

Metal ions significantly affected the enzymatic catalytic activity based on the above results. Endoglucanase of GH8 family has two conserved glutamate residues at the active site and the cellulase activity might be restrained when metal ions is bound to the radical of these residuals [22]. Mg^2+^ might enhance cellulase activity through altering the dimensional structure of BcsZ or stabilizing the enzyme structural conformation. Co^2+^, Mn^2+^, and Fe^2+^ enhanced the enzyme activity of GHF9 endoglucanase from *Reticulitermes speratus*, but Pb^2+^ and Cu^2+^ inhibited its enzyme activity [23]. Meleiro et al cloned and overexpressed an endoglucanase (Egst) from *Scytalidium thermophilum* that showed high catalytic activity at harsh condition. NH^4+^, Na^+^, K^+^, Ba^2^+, Ca^2+^ and Mg^2+^ had little effect on Egst. Cu^2+^ presented a slight inhibitory effect and Hg^2+^ had a strong inhibitory effect on Egst [24].

BcsZ could hydrolyze CMC-Na, RAC and glucan. BcsZ had a higher affinity for CMC than Thcel9A (12.02 mg/mL) from *Thermobifida halotolerans* YIM 90462 T [25], the purified endoglucanase (21.01 mg/mL) from *Aspergillus niger B03* [26] and C67–1 (37 mg/mL) from metagenomes of buffalo rumens [27]. It had a weaker affinity for CMC than the endoglucanase from *Bacillus sp.* (0.8 mg/mL) [28]. The V_max_ value (0.3 μM/min·mg) of BcsZ was lower than Cell-1 (0.84 μmol/min·mg) [29] and an endoglucanse (1000 μM/min) [28]. The V_max_ value of BcsZ was low indicating this enzyme had a weak catalytic efficiency for CMC.

The BcsZ from *E. coli* ZH-4 could be classified as an endo-β-1, 4 glucanase according to its product pattern. Some other endo-β-1, 4 glucanases also have the above properties. An endoglucanase was expressed and characterized from *Serratia proteamaculans* CDBB-1961 by Cano-Ramírez, and then was applied to hydrolyze CMC to glucose and cello oligosaccharides [30]. The endo-β-1, 4 glucanase (E4–90) from *Termomonospora fusca* hydrolyzed CMC to produce cellobiose, cellotriose, cellotetraose and glucose [31]. As the previous study, a purified β-1, 4 endoglucanase (LbGH5) could hydrolyze CMC and phosphoric acid swollen cellulose (PASC) with the enzymatic hydrolysate including cellobiose, cellotriose, cellotetraose and a small amount of cellopentaose and glucose [32]. In contrast, a thermophilic endo-1, 4-β- glucanase from *Sulfolobus shibatae* hydrolyzed cellotetraose and cellopentose not cellobiose or cellotriose. The products of CMC hydrolysis were cellobiose, cellotriose, cellotetraose and cellopentose [33]. Cellobiose and cellotriose was produced from PASC and Avicel respectively, and cellobiose, cellotriose and cellotetraose were produced from CMC with Endo-glucanase EG5C-1 [34]. The release of significant amount of glucose also explained why *E.coli* ZH-4 can grow on cellulose. Our experiments also showed that expression of BcsZ in *E.coli* BL21 resulted in the growth of *E.coli* BL21 on cellulose. These identification and characterization of BcsZ may improve understanding the adaptation of *E. coli* to different environment.

## Conclusions

BcsZ was shown to be responsible for extracellular endo-glucanase activity and cellulolytic capability of *E. coli* ZH-4. Expression, purification and characterization of the endo-glucanase BcsZ showed that it was thermotolerant and pH tolerant. This enzyme could hydrolyze CMC to produce glucose, cellobiose, cellotriose and unknown cello oligosaccharides, which explained why *E. coli* ZH-4 can grow on cellulose.

## Methods

### Bacterial strains and culture conditions

The cellulolytic *E. coli* ZH-4 was isolated from the bovine rumen by our laboratory and it was preserved in China General Microbiological Culture Collection Center (CGMCC) (Preservation No. 12427). *E. coli* DH5α was used for plasmid amplification, and *E. coli* BL21 (DE3) was used as the host for recombinant protein expression with pET28a as vector. *E. coli* strains were routinely cultured in Luria–Bertani (LB) medium at 37 °C with shaking at 220 rpm. Kanamycin was added when needed at a final concentration of 50 μg/mL.

### Quantitative RT-PCR analysis

Total RNA was extracted using the RNAprep pure Cell/Bacteria Kit (TIANGEN) following manufacturer’s instruction. The concentration and quality of RNA was measured using the Nano-Drop spectrophotometry (NanoDrop Technologies, Wilmington, DE, USA). The cDNA was synthesized according to the First Stand cDNA Synthesis Kit (TOYOBO) following the manufacturer’s protocol. The primers were displayed in Table 2. The PCR reaction included 12.5 uL SYBR Green Realtime PCR Master Mix (TOYOBO), 2.5 uL diluted cDNA (500 mM) reaction mixture, 1uL each forward and reverse primer (10 umol) and 8.0 uL ddH_2_O. The RT-PCR assays were performed on a 7900HT Fast Real-Time PCR System (Applied Biosystems, Carlsbad, California, USA). The relative gene expression calculated by the equation of 2^(−ΔΔCt)^ method (Applied Biosystems Research Bulletin No. 2 P/N 4303859).

**Table 2:** The primers of qRT-PCR

Primers	Sequences (5′-3′)
*bcsZ*-F	GAGAACAGTAAGTGGGAAGTGC
*bcsZ*-R	AACGCTGCTCTTTCCACAAACG
16S rRNA-F	GCTCAACCTGGGAACTGC
16S rRNA-R	CCACGCTTTCGCACCTGA

### Western blot analysis

Western blotting was performed as described by Sambrook [35]. Proteins separated by SDS-PAGE were transferred onto polyvinylidene difluoride (PVDF) membranes (Millipore, Billerica, MA, USA) using the Bio-Rad semi-dry apparatus. The blots were incubated with primary anti-BcsZ serum in 1:2000 dilution in 2% (wt/vol) skimmed milk for 1 h with agitation at room temperature followed by washes with PBST buffer (PBS buffer with 1% Tween) for 3 times. The secondary antibody with horseradish peroxidase (HRP) conjugated goat anti-rabbit (1:2000 dilution) was performed as above. Blots were developed with the ECL Plus Kit (Thermo Scientific, US) following the manufacturer’s directions. The in-gel identification of secreted protein by mass spectrometry analysis was performed by Shenzhen BGI gene co., LTD.

### Sequence analysis

*bcsZ* gene sequences from *E. coli* ZH-4 and *E. coli* MG1655 was compared using the BLASTN program (https://blast.ncbi.nlm.nih.gov/Blast.cgi).

### Plasmid construction

To overexpress BcsZ, *bscZ* gene was amplified from *E. coli* ZH-4 using primers BcsZ-F (5′- CGCGGATCCGGGTGTGAATTTGCGCATTCCT-3′) and BcsZ-R (5′-CATGCCATGGGCAATGTGTTGCGTAGTGGAAT-3′). The PCR products were then digested and cloned into *Bam*HI and *Nco*I sites of pET-28a plasmid. His-tag was located in C-terminus of *bcsZ*. The constructed expression vector pET-28a-BcsZ was verified by DNA sequencing.

### Protein expression and purification

Overnight culture of *E. coli* BL21 (DE3) harboring plasmid pET-28a-BcsZ was inoculated to fresh LB medium supplemented with 50 μg/mL kanamycin in 1:100 dilution and cultured at 37 °C with shaking. The *E. coli* BL21 (DE3) carrying the empty plasmid was used as control. To induce *bscZ* gene expression, isopropyl-β- d-thiogalactoside (IPTG) was added to the culture at a final concentration of 0.25 mM when the optical density at 600 nm reached 0.4~0.6. Cells were harvested by centrifugation (8000×g, 10 min) after 24 h cultivation at 25 °C with shaking at 220 rpm. The resulting cells were suspended in 10 mM phosphate buffer saline (PBS) with PMSF and DNase I. The cells were lysed using JNBIO JN-3000 PLUS high-pressure cell press. Then, the crude cell lysate was prepared by centrifugation (12,000×g, 30 min) to remove the cell debris.

The BcsZ was further purified by affinity chromatography using HisPur Cobalt Resin (Thermo Fisher Scientific Inc) according to the manufacturers’ instruction. The purity and homogeneity of the purified enzyme was detected by SDS–PAGE.

### Enzyme assay and protein determination

The enzyme activity of BcsZ was determined by the standard DNS method [36]. The mixture of 1.5 mL 1% (w/v) CMC solubilized in 50 mM phosphate buffer (pH 6.0) and 0.5 mL purified BcsZ was incubated at 50 °C for 30 min. The the reaction was stopped by the addition of 3 mL of DNS. The release of reducing sugars was determined by the absorbance at 540 nm. One unit of activity is defined as the amount of enzyme that released 1 μmol of reducing sugars/min from CMC. Protein concentration was measured by Bradford method using bovine serum albumin as standard [37].

### Basic biochemical character of BcsZ

The optimum pH was determined by measuring BcsZ activity in different buffers (50 mM) of pH ranging from 4.0 to 10.0: citric acid buffer for pH 4.0–6.0; phosphate buffer for pH 6.0–8.0; Glycine-NaOH buffer for pH 8.5–10.0. The optimum temperature was identified by incubating the enzyme in phosphate buffer (pH 6.0) at different temperatures (30–80 °C). For thermostability determination of BcsZ, it was incubated at different temperatures 4, 40, 50, 60 and 70 °C for 0.5, 1, 2, 3, 4, 5, 10 and 24 h. The residual endoglucanase activity was determined, respectively. The influence of metal ions on enzyme activity of BcsZ was measured in presence of tested metal ions at indicated concentration. All enzyme activity was measured as previously described. The highest enzymatic activity was used as benchmark of 100% activity when calculating relative activity.

### Kinetic constants

The kinetic parameter values Michaelis-Menten constants (K_m_) and maximum velocity (V_max_) were determined to calculate the K_m_ value of BcsZ on CMC hydrolysis according to double-reciprocal Lineweaver–Burk plots (Eq. 1). The activity assay was performed with CMC at different concentrations at pH 6.0 and 50 °C for 10 min.

1 $$ \frac{1}{V}=\frac{K_m}{V_{max}}\ \frac{1}{\left[S\right]}+\frac{1}{V_{max}} $$

### Substrate specificity

The hydrolytic ability of BcsZ on 1% (w/v) of various substrates were determined by DNS method under optimal conditions. The substrate included Avicel, CMC-Na, regenerated amorphous cellulose (RAC), xylan, cellobiose, laminarin and chitin.

### Thin layer chromatographic (TLC)

The products were analyzed by Thin Layer Chromatographic (TLC) as previously described when BcsZ hydrolyzed CMC and RAC [38].

### Statistical analysis

All experiments were conducted in triplicate and the data were presented as mean values ± standard deviation.

## Data Availability

The datasets generated and/or analyzed during the current study are available on the GenBank repository, https://www.ncbi.nlm.nih.gov/nuccore/KY965823. The GenBank accession number for the nucleotide sequence of *bcsZ* referred to in the text is KY965823. Other datasets presented in the article and are available from the corresponding author upon reasonable request.

## Abbreviations

CGMCC: China General Microbiological Culture Collection Center
CMC: carboxymethylcellulose
DNS: dinitrosalicylic
GH: Glycoside hydrolase
HRP: horseradish peroxidase
IPTG: isopropyl-β- d-thiogalactoside
LB: Luria–Bertani
PBS: phosphate buffer saline
PVDF: polyvinylidene difluoride
RAC: Regenerated amorphous cellulose
SDS–PAGE: Sodium dodecyl sulfate–polyacrylamide gel electrophoresis
TLC: Thin layer chromatographic
